# An unusual presentation of cutaneous sarcoidosis induced by Koebner’s phenomenon

**DOI:** 10.1093/omcr/omaf147

**Published:** 2025-08-25

**Authors:** Maryam Ghaleb, Ouiame El Jouari, Salim Gallouj

**Affiliations:** Dermatology Department, Mohammed VI University Hospital, Faculty of Medicine and Pharmacy, Abdelmalek Essaâdi University, Tangier, 90000, Morocco; Dermatology Department, Mohammed VI University Hospital, Faculty of Medicine and Pharmacy, Abdelmalek Essaâdi University, Tangier, 90000, Morocco; Dermatology Department, Mohammed VI University Hospital, Faculty of Medicine and Pharmacy, Abdelmalek Essaâdi University, Tangier, 90000, Morocco

**Keywords:** cutaneous sarcoidosis, Koebner phenomenon, granulomatous dermatoses, systemic sarcoidosis, Hydroxychloroquine

## Abstract

We report an unusual case of cutaneous sarcoidosis presenting as papulonodular lesions strictly localized to the nasal bridge and medial canthus—corresponding to areas of pressure from eyeglass frames. This rare distribution suggests the involvement of Koebner’s phenomenon, a form of reactive isomorphism rarely reported in sarcoidosis. The cutaneous lesions, present for four years, led to dermatological evaluation and subsequent histopathological confirmation of sarcoidosis. Biological and radiological investigations further revealed pulmonary involvement, establishing the diagnosis of systemic sarcoidosis. The patient was treated with hydroxychloroquine and intra-lesional corticosteroids, showing marked improvement after three months and no recurrence at six months. This case underscores the importance of recognizing atypical presentations of cutaneous sarcoidosis, which may serve as an early clinical gateway to the diagnosis of systemic disease.

## Introduction & Objectives

Cutaneous sarcoidosis is a dermatological manifestation of systemic sarcoidosis, a multisystem granulomatous disease characterised by non-caseating epithelioid granulomas. The Koebner phenomenon, also known as the ischemic response, is characterised by the manifestation of lesions that are characteristic of a specific disease in areas of the body that have sustained injury or mechanical stress in individuals who are genetically susceptible to the development of such lesions. Although traditionally linked to conditions such as psoriasis and lichen planus, this phenomenon has also been documented in cutaneous sarcoidosis [1, 2].

In the context of sarcoidosis, the Koebner phenomenon manifests as the development of lesions on pre-existing scars, tattoos, or areas subjected to chronic mechanical pressure. The development of sarcoid lesions on surgical scars, burns, and injection sites has been reported [3, 4].

The present case report documents a distinctive instance of cutaneous sarcoidosis, manifesting as papulonodular lesions strictly confined to the nasal bridge and medial canthus. These anatomical regions are subject to continuous pressure from eyeglass frames. This presentation is noteworthy for its unusual nature, which highlights a rare Koebner phenomenon and illustrates how localized mechanical stress may unmask an underlying systemic disease.

## Case report

**Figure 1:**
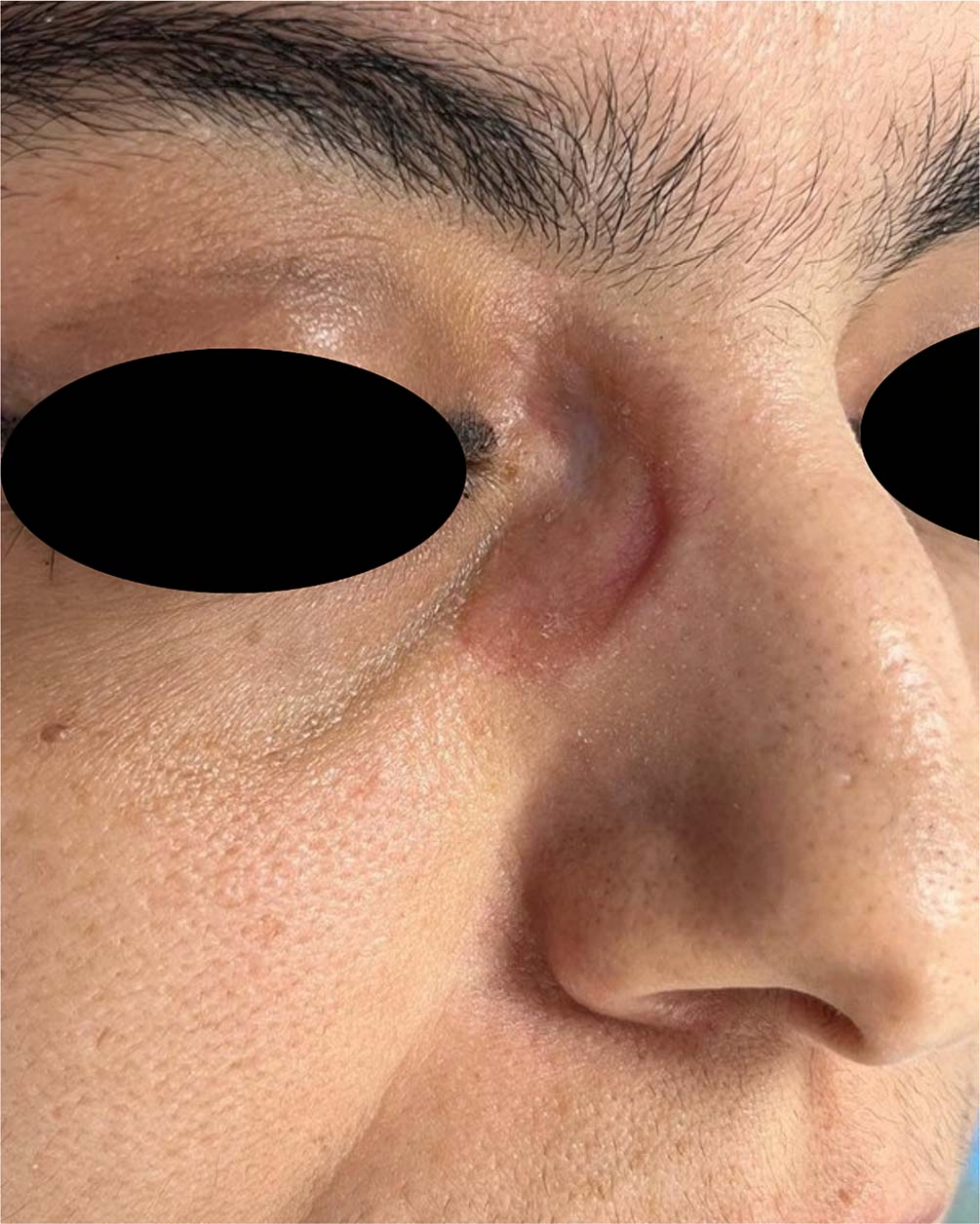
Papulonodular cutaneous sarcoidosis presenting as erythematoviolaceous, infiltrated lesions on the nasal bridge and medial canthus, corresponding to eyeglass pressure points.

**Figure 2:**
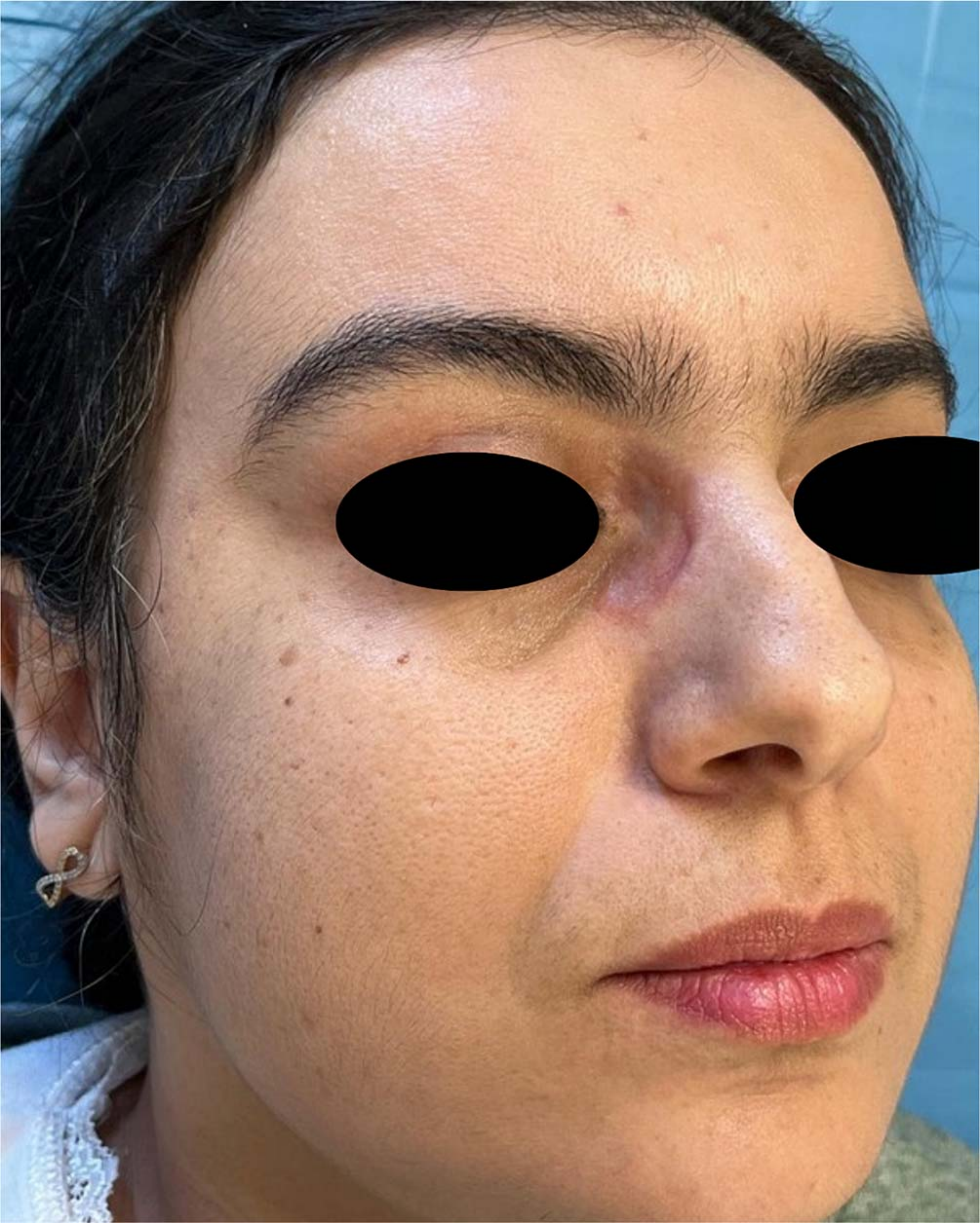
Papulonodular cutaneous sarcoidosis presenting as erythematoviolaceous, infiltrated lesions on the nasal bridge and medial canthus, corresponding to eyeglass pressure points.

**Figure 3:**
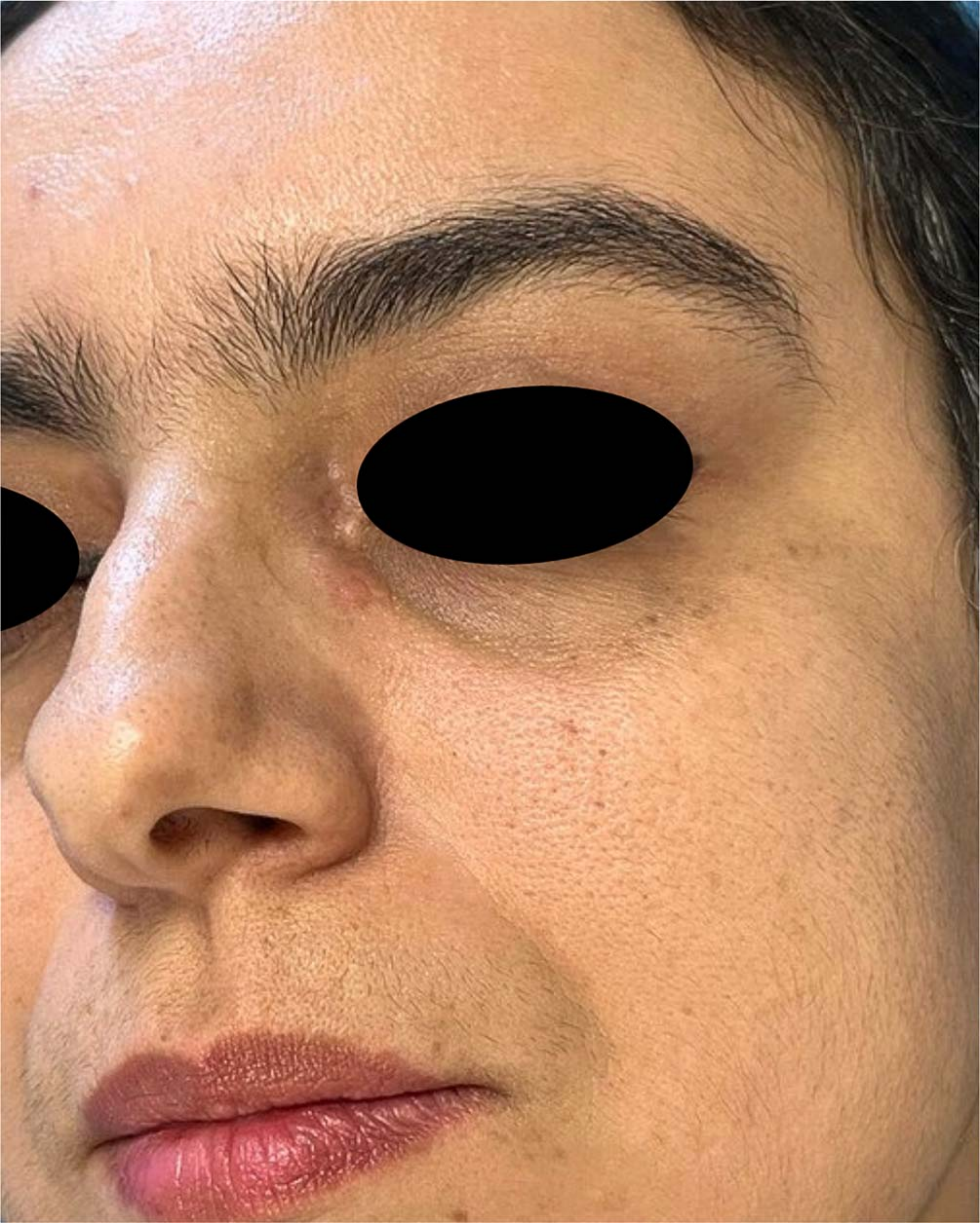
Papulonodular lesion of cutaneous sarcoidosis on the contralateral side.

The present case study concerns a 40-year-old female patient who initially presented to the dermatology department for skin lesions that had been evolving over a period of 4 years. A detailed dermatological examination revealed the presence of erythematoviolaceous, infiltrated, painless, non-pruritic, and confluent papulonodular lesions, with the largest measuring 2 cm. The lesions were bilateral, symmetrical, and located at the root of the nose, with extension towards the internal canthus (Figs 1–3).

**Figure 4:**
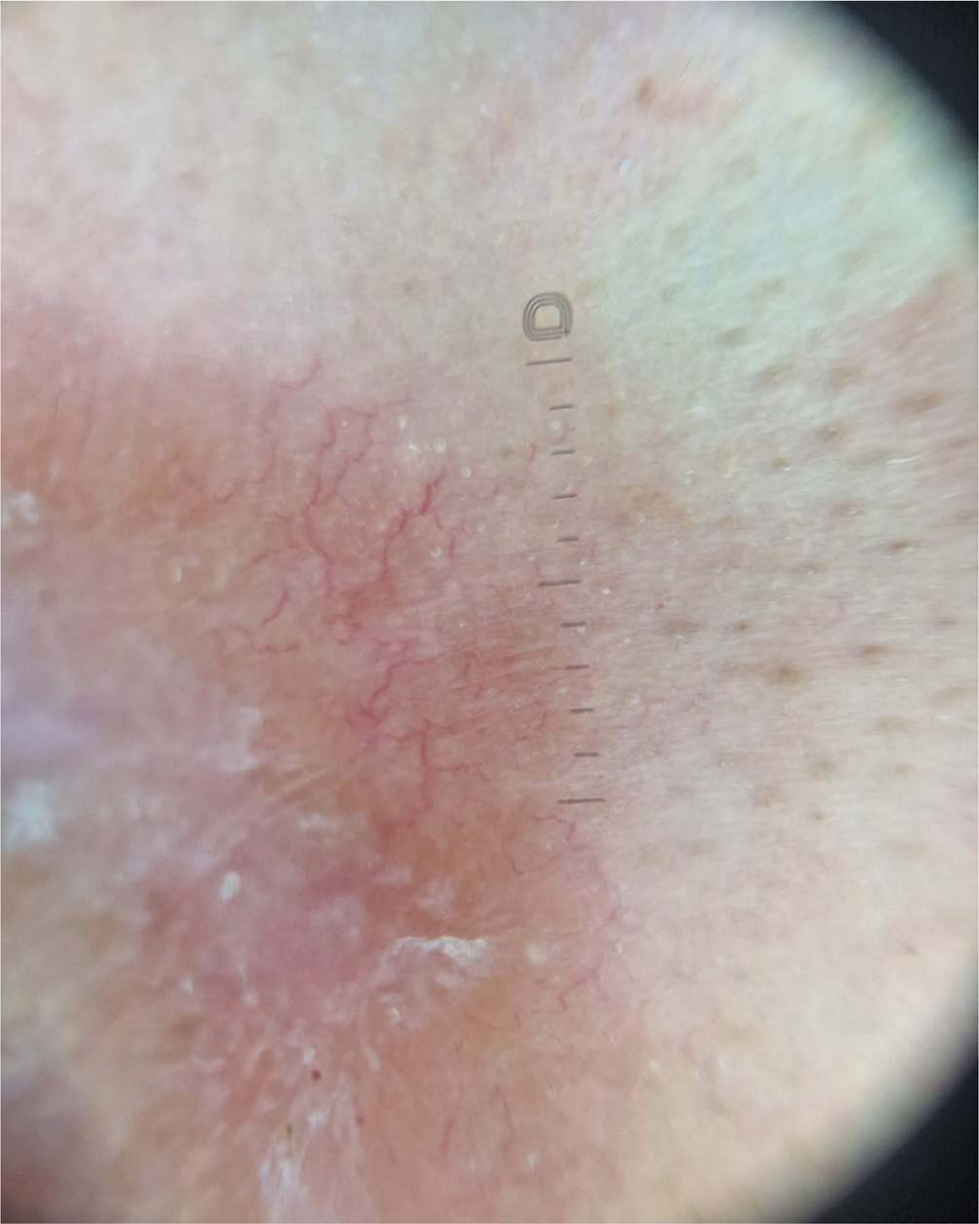
Orange areas accompanied by linear arborizing vessels.

Dermoscopy revealed orange areas accompanied by linear arborizing vessels (Fig. 3). A skin biopsy was performed, which revealed the presence of follicular granulomatous dermatitis with epithelioid giant cells (Fig. 4), thereby confirming the diagnosis of cutaneous sarcoidosis. Consequently, a diagnosis of cutaneous sarcoidosis was established.

**Figure 5:**
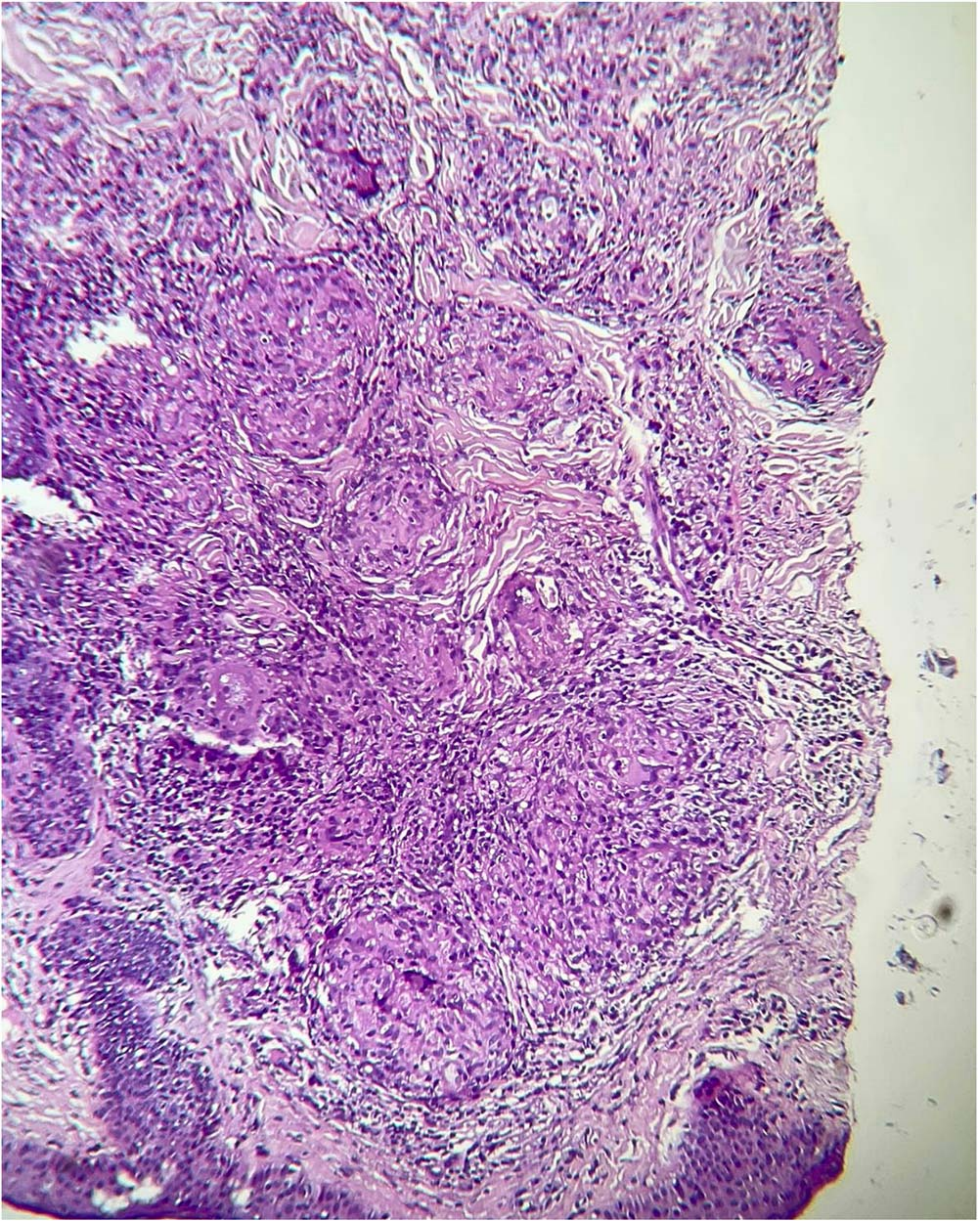
Follicular granulomatous dermatitis with epithelioid giant cells.

Further analysis revealed a significant elevation of angiotensin-converting enzyme (140 U/l; reference range: 20–70 U/l). Chest radiography, followed by a chest CT scan, was performed and revealed features indicative of stage 2 mediastinal-pulmonary sarcoidosis. Subsequently, the patient exhibited respiratory symptoms, which prompted a referral to the pulmonology department for further evaluation and management.

The diagnosis of systemic sarcoidosis, with both cutaneous and pulmonary involvement, was established on the basis of clinical, biological, histological, and radiological findings. The treatment regimen comprised synthetic antimalarial drugs (APS), specifically Plaquenil at a dose of two tablets per day, in combination with intra-lesional corticosteroid injections.

The patient exhibited a favorable response to the treatment, with a significant improvement in the lesions evident after three months of therapy. After a period of six months had elapsed, no additional clinical, biological, or radiological evidence of sarcoidosis had been identified. Nevertheless, a more extensive follow-up protocol is required to evaluate the likelihood of recurrence.

## Discussion

Cutaneous sarcoidosis is frequently referred to as the ‘great imitator’ due to the variability of its clinical manifestations, which can encompass macules, papules, nodules, plaques, lupus pernio, or scar sarcoidosis. While not specific, these lesions may offer a crucial diagnostic clue, especially in the absence of systemic symptoms [3, 5].

Koebner’s phenomenon, which was first described in cases of psoriasis, pertains to the manifestation of lesions in response to trauma or mechanical stress in susceptible individuals. While it is most commonly associated with conditions such as psoriasis, lichen planus, and vitiligo, Koebner’s phenomenon has also been reported, albeit infrequently, in granulomatous diseases such as sarcoidosis [1, 2, 6].

In the present case, the localisation of lesions strictly to the nasal bridge and medial canthus, in direct contact with the eyeglass frames, suggests a mechanical triggering of cutaneous sarcoidosis by chronic pressure. This presentation aligns with previously documented, albeit rare, examples of sarcoid lesions developing at the sites of trauma, surgical scars, tattoos, or injection sites [2, 4, 7, 8].

The observations made in this study are noteworthy for two particular features. Firstly, it is important to note that the cutaneous reaction occurred after a delay of over four years. Secondly, the precise anatomic distribution of the lesions corresponded to the mechanical zones of contact with the eyeglass frames. This presentation is noteworthy for its unusual illustration of how Koebner’s phenomenon may act as a revealing factor for systemic sarcoidosis [6].

The diagnosis of pulmonary involvement was confirmed by biological markers, including elevated angiotensin-converting enzyme levels, and imaging studies, which also supported the systemic nature of the disease. Histopathological analysis remains paramount for definitive diagnosis, with the presence of non-caseating epithelioid granulomas in the dermis being a hallmark finding [3, 9].

Biological markers, such as elevated angiotensin-converting enzyme levels, and imaging studies supported the diagnosis of pulmonary involvement, confirming the systemic nature of the disease. Histopathological analysis remains paramount for definitive diagnosis, with the presence of non-caseating epithelioid granulomas in the dermis being a hallmark finding [3].

The patient demonstrated a favourable response to initial treatment with hydroxychloroquine and local corticosteroid injections, aligning with contemporary management strategies for cutaneous sarcoidosis [10]. However, further discussion of additional therapeutic options, including systemic corticosteroids, methotrexate, or TNF-α inhibitors in cases of resistance, would enrich the clinical perspective [10, 11]. Furthermore, the incorporation of a more detailed description and comparative analysis of the dermoscopic and histopathologic images would enhance diagnostic clarity.

It is important to note that this report does not include a dedicated literature review. The integration of references to analogous reported cases of Koebner-induced sarcoidosis in the extant literature would serve to reinforce the scientific context and highlight the relevance of the present case [4, 7, 8].

Finally, the identification of potential risk factors—such as genetic predisposition, environmental triggers, or immunological alterations—could provide insights into the patient’s heightened susceptibility to Koebner’s phenomenon, and thus should be addressed [12].

## Conclusion

In conclusion, this case illustrates an uncommon presentation of cutaneous sarcoidosis induced by localized mechanical pressure from eyeglass frames, consistent with Koebner’s phenomenon. The diagnosis was confirmed through histopathological examination and radiological findings, which also revealed systemic involvement. The patient exhibited a positive response to treatment with Plaquenil and intra-lesional corticosteroid injections, demonstrating significant clinical improvement after a period of three months and no recurrence after a period of six months. This case underscores the importance of recognising atypical cutaneous presentations, which can serve as early indicators of systemic sarcoidosis, allowing for timely diagnosis, appropriate therapeutic intervention, and close follow-up. It is recommended that future reports consider incorporating a more detailed image analysis, an expanded discussion of treatment alternatives, consideration of patient risk factors, and a review of analogous cases, with a view to enhancing the contribution to the existing body of literature.
